# Methodological Limitations in Determining Astrocytic Gene Expression

**DOI:** 10.3389/fendo.2013.00176

**Published:** 2013-11-25

**Authors:** Liang Peng, Chuang Guo, Tao Wang, Baoman Li, Li Gu, Zhanyou Wang

**Affiliations:** ^1^Department of Clinical Pharmacology, China Medical University, Shenyang, China; ^2^Institute of Neuroscience, College of Life and Health Sciences, Northeastern University, Shenyang, China

**Keywords:** astrocyte culture, BAC transgenic animals, fluorescence-assisted cell sorting, GFAP, immunohistochemistry, *in situ* hybridization

## Abstract

Traditionally, astrocytic mRNA and protein expression are studied by *in situ* hybridization (ISH) and immunohistochemically. This led to the concept that astrocytes lack aralar, a component of the malate-aspartate-shuttle. At least similar aralar mRNA and protein expression in astrocytes and neurons isolated by fluorescence-assisted cell sorting (FACS) reversed this opinion. Demonstration of expression of other astrocytic genes may also be erroneous. Literature data based on morphological methods were therefore compared with mRNA expression in cells obtained by recently developed methods for determination of cell-specific gene expression. All Na,K-ATPase-α subunits were demonstrated by immunohistochemistry (IHC), but there are problems with the cotransporter NKCC1. Glutamate and GABA transporter gene expression was well determined immunohistochemically. The same applies to expression of many genes of glucose metabolism, whereas a single study based on findings in bacterial artificial chromosome (BAC) transgenic animals showed very low astrocytic expression of hexokinase. Gene expression of the equilibrative nucleoside transporters ENT1 and ENT2 was recognized by ISH, but ENT3 was not. The same applies to the concentrative transporters CNT2 and CNT3. All were clearly expressed in FACS-isolated cells, followed by biochemical analysis. ENT3 was enriched in astrocytes. Expression of many nucleoside transporter genes were shown by microarray analysis, whereas other important genes were not. Results in cultured astrocytes resembled those obtained by FACS. These findings call for reappraisal of cellular nucleoside transporter expression. FACS cell yield is small. Further development of cell separation methods to render methods more easily available and less animal and cost consuming and parallel studies of astrocytic mRNA and protein expression by ISH/IHC and other methods are necessary, but new methods also need to be thoroughly checked.

## Introduction

Enzymes and transporters, involved in production and degradation of glutamate and GABA are expressed in astrocytes. Some have been known for a long time to be astrocyte-specific, e.g., glutamine synthetase (GS) (1), pyruvate carboxylase (2), and cytosolic malic enzyme (3). However, identification of either mRNA or protein expression in astrocytes in the brain *in vivo* or intact brain tissue, such as brain slices, is difficult on account of its extreme anatomical complexity. Conventional immunohistochemical methods seem occasionally to fail in demonstrating the expression of certain genes, as recently demonstrated in the case of aralar, a glutamate/aspartate exchanger operating in the malate-aspartate-shuttle (MAS). Its abundant mRNA (4) and protein (5) expression has been demonstrated by usual biochemical methods in freshly isolated mouse astrocytes and in well differentiated cultured astrocytes (5). However, gene expression of the aralar gene has repeatedly been found to be absent or sparsely expressed when morphology-based immunochemical methods were used, as will be described in Section “Determination of the Expression of Genes Involved in Different Pathways using Different Methodologies.”

Traditional morphology-based methods for studying cell type-specific gene expression in brain *in vivo* or in excised brain tissues are *in situ* hybridization (ISH) and immunohistochemistry (IHC), analyzing mRNA and protein expression, respectively. Immunocytochemistry (ICC) is used for the same purpose in isolated cells. A technique, which has been newly established for adult brain, fluorescence-assisted cell sorting (FACS) yields highly purified populations of different types of brain cells (4, 6). It uses insertion of a fluorescent compound into the cell by aid of the promoter of an astrocyte-specific gene, such as glial fibrillary acidic protein (GFAP) or S100β, a principle developed for insertion of green fluorescent protein (7), and thus depends upon preservation of an intact cell. This and related methods are often used for determination of mRNA expression by microarray analysis, a quantitatively less accurate method, as evident from Tables 1 and 2, where different results quite often were obtained by different authors and from results by Hertz et al. (8), where consistency between different samples in some cases was poor. Nevertheless it is useful because of its requirement for very little tissue. This is a necessity for simultaneous determination of the expression of multiple genes, since the cell yield by FACS is small. The microarray analysis provides numbers, not a direct indication whether a specific gene is expressed or not. Therefore a numerical analysis is needed. In the paper by Lovatt et al. (4) the authors interpret several results as indicating whether a gene is expressed or not, but in their published Table (9) only numbers are presented. In reference (9), they also present a Table showing a comparison with non-astrocytes in the case of Lovatt et al. (4), and neurons in the case of Cahoy et al. (6) and indicate fold-enrichment and *P* values. This table has been used in connection with the Lovatt (4) and Doyle (10) data in Tables 1 and 2 in the present paper, whereas the Cahoy data is based upon the numbers in the comprehensive Table provided by (6). This is because astrocyte expression, not enrichment is the topic of this paper.

**Table 1:** **Expression of astrocytic genes determined by different methodologies**.

Protein title	Gene	mRNA				Protein		Gene
		*In situ* hybridization	FACS RT-PCR	Microarray	Culture RT-PCR	Immuno-histochemistry	Culture	
Aralar	*Slc25a12*		Present (5)	+ (4) + (6) + (10)	Present (5)	Absent (11, 12)	Present (5)	*Slc25a12*
						Present (13)	
GLAST	*Slc1a3*	Present (14, 15)		+ (4) + (6) + (10)		Present (16, 17)	Present (18)	*Slc1a3*
GLT-1	*Slc1a2*	Present (19, 20)		+ (4) + (6) + (10)		Present (21)	Present (22, 23)	*Slc1a2*
GAT1	*Slc6a1*	Present (24, 25)		+ (4) + (6) + (10)		Present (26)		*Slc6a1*
GAT 2	*Slc6a13*	Present (24, 26)		− (4) − (6) − (10)		Present (26, 27)		*Slc6a13*
GAT 3	*Slc6a11*	Present (26, 27)		+ (4) + (6) + (10)		Present (28, 29)		*Slc6a11*
BGT 1	*Slc6a12*			− (4) − (6) − (10)	Present (30, 31)			*Slc6a12*
ENT1	*Slc29a1*	Present (32, 33)	Present (34)	− (4) + (6) + (10)	Present (35)			*Slc29a1*
ENT2	*Slc29a2*	Present (32)	Present (34)	− (4) + (6) + (10)	Present (35)			*Slc29a2*
ENT3	*Slc29a3*		Present (34)	− (4) + (6) + (10)	Present (35)			*Slc29a3*
ENT4	*Slc29a4*		Present (34)	− (4) − (6) + (10)		Absent (36)		*Slc29a4*
CNT1	*Slc28a1*		Absent (34)		Present (37)			*Slc28a1*
CNT2	*Slc28a2*	Absent (33)	Present (34)	− (4) − (6) + (10)	Present (35)			*Slc28a2*
CNT3	*Slc28a3*	Low expression (33)	Present (34)	− (4)	Absent (35)			*Slc28a3*
Na,K-ATPase α1	*Atp1a1*	Present (38–40)		− (4) + (6) − (10)		Present (41)	Present (41)	*Atp1a1*
Na,K-ATPase α2	*Atp1a2*	Present (40, 42)	Present (43)	+ (4) + (6) + (10)		Present (41)	Present (41)	*Atp1a2*
NKCC1	*Slc12a2*	Present (42, 44, 45)		− (4) + (6) + (10)			Present (46)	*Slc12a2*

*The data for aralar, Na,K-ATPase, and the nucleoside transporters are supposed to be comprehensive, whereas those for transporters involved in glutamate/GABA turnover are rather representative, at least with respect to immunohistochemistry and *in situ* hybridization. Genes consistently found to be expressed by all methodologies are shown in blue, those only found absent in one study in green, and results from studies using FACS-isolated astrocytes in red. As described in the text, microarray data were assigned as either positive or negative based on the number values in the microarray analysis itself [(6) http://www.stanford.edu/group/exonarray/cgi-bin/plot_selector.pl] and fold-enrichment and significance [6] and [10] as shown by Lovatt and Nedergaard (9), but using less stringent criteria with respect to fold enrichment (≥1.5) than in the original publications. This was found to be justifiable, because the microarray data here are used together with other data, but in the original publications as the only indication of gene presence. Reference numbers given in this Table refer to those in the reference list*.

**Table 2:** **Drug effects on gene expression and editing in astrocytes are identical in freshly isolated cells from treated animals and primary cultures of astrocytes, but the expression is often not recognized by the microarray analyses, indicatest as + or −**.

Protein	Gene	Drug	FACS	Culture	Microarray			
					(4)	(6)	(10)	[4*]
5-HT_2B_ receptor expression	*Htr2b*	Fluox	Up	Up	−	−	−	
5-HT_2B_ editing	*Htr2b*	Fluox	Up	Up				
5-HT_2c_ receptor expression	*Htr2c*	Fluox	Unchanged	Unchanged	−	−	−	
cPLA_2a_	*Pla2g4a*	Fluox	Up	Up	−	−	−	+
sPLA_2_	*Pla2g2a*	Fluox	Unaltered	Unaltered	−	−	−	
ADAR2	*Adarb1*	Fluox	Up	Up	−	−	−	−
GluK2 expression	*Grik2*	Fluox	Up	Up	+	+	−	−
GluK2 editing	*Grik2*	Fluox	Up	Up				
GluK4 expression	*Grik4*	Fluox	Unchanged	Unchanged	−	−	−	−
cfos expression	*cFos*	Fluox	Up	Up	−	+	−	+
fosB expression	*Fosb*	Fluox	Up	Up	−	+	−	+
NBCe1	*Slc4a4*	Cbz	Up	Up	+	+	+	+
GluK2	*Grik2*	Cbz	Down	Down				
cPLA_2_	*Pla2g4a*	Cbz	Up	Up				

The Table shows all experiments in which we have compared drug [fluoxetine (Fluox) or carbamazepine (Cbz)] effects in cultured astrocytes and in astrocytes obtained by FACS. Complete agreement was found between these two preparations, but the correlation was poor with expression of the same genes obtained using microarray analysis and with the cultures, different from ours, studied by Cahoy et al. using microanalysis (4*). The FACS and culture data are from (47) and (48). Microarray data show only gene expression and are from Lovatt et al. [4], Cahoy et al. [6] and Doyle et al. [10] Cahoy et al. (6) also presented data for cultures different from ours, shown under [4]. As in Table 1, expression is shown as either present (+) or absent (−) based on the number values in the microarray analysis itself [2 and 4] or fold-enrichment and significance [1] and [3] shown by Lovatt and Nedergaard (9).

The microarray analysis by Doyle et al. (10) also provides numbers. However, their analysis was not based on FACS but was based on generation of transgenic mice that expressed the ribosomal protein L10a in a cell-specific manner. Since this protein had been tagged with enhanced green fluorescent protein (EGFP), they could check proper cell-specific labeling by the green fluorescence and simultaneously use anti-GFP antibodies for immunopurification to enrich ribosome-associated, actively translated mRNAs. This procedure allowed them to bypass the cell separation procedure and instead use a total cell homogenate. The astrocytic-specific *Aldh1L1* (6) was used for their studies of astrocytic gene expression. The methodology employed in each of these studies is described in more detail in Section “Cell Sorting Based on Recognition of Cell-Specific Proteins.” Here it suffices to note that in spite of the different procedures employed in these three studies, and although Lovatt et al. (4) and Doyle et al. (10) used adult mice, and the oldest mice studied by Cahoy et al. (6) were 17-day-old, the results are relatively, although far from completely, similar. In Table 1 the two methods are therefore indicated close to each other, with all microarray results in red.

If only expression of a single or a few genes are analyzed, FACS-isolated cells can also be used for a more accurate determination of mRNA by reverse transcription-polymerase chain reaction (RT-PCR), requiring larger cell samples, and even of protein by Western blot followed by reaction with a specific antibody, needing still more tissue. Gene expression in primary cultures of astrocytes has also been utilized as a method to determine astrocytic characteristics. Some recent papers criticizing this technique (49, 50) have unjustifiably failed to recognize that many types of astrocytic cultures exist and that they are not all identical. The cultures used by ourselves differ vastly both in methodology and characteristics from those previously used by Harold Kimelberg and from those discussed by Foo et al. (50). In contrast to those used by Foo et al. (50) they are grown in the presence of serum and they are differentiated by treatment with dibutyryl cyclic AMP (dBcAMP), necessary for the development of certain specific features, e.g., K^+^-induced glycogenolysis (51) a well established phenomenon in intact brain tissue (52). In contrast to many other astrocyte cultures prepared from rats they are obtained from mice (53). Peculiarly enough, this does make a difference (54), e.g., in membrane conductance, which these authors found comparable only in mouse cells with the high membrane conductance characteristic of astrocytes in the brain *in vivo*.

The discrepancy regarding expression of the aralar gene between freshly isolated astrocytes and those studied in intact tissue by IHC raises the question whether expression of other important genes may be underestimated in the literature. This will be discussed in the present review by a comparison between results of determination of astrocytic gene expression in intact tissues, freshly isolated cells and cultures, presented in Section “Determination of the Expression of Genes Involved in Different Pathways using Different Methodologies.” Some additional enzymes involved in glucose metabolism will be discussed in Section “Expression of Genes of Enzymes Catalyzing Glucose Metabolism,” a few of those involved in the glutamate-glutamine cycle in Section “Enzymes and Transporters Operating in Glutamate and GABA Turnover,” several of those involved in astrocytic ATP signaling (nucleoside transporter and adenosine kinase expression) in Section “Nucleoside Transporters and Adenosine Kinase,” and those involved in K^+^ homeostasis in Section “Expression of Genes of Transporters Involved in K^+^ Clearance from the Extracellular Space.” The two former are directly relevant for the present Research Topic. The two latter represent other important astrocytic functions, and are discussed, although more briefly, to better evaluate potential failures of some methodologies to disclose astrocytic gene expression. These sections will be followed by more detailed descriptions of individual methodologies in Section “Description of Methodologies Used for Determination of Gene Expression.”

## Determination of the Expression of Genes Involved in Different Pathways using Different Methodologies

### Expression of genes of enzymes catalyzing glucose metabolism

#### Lack of astrocytic aralar expression in immunohistochemical studies versus prominent mRNA and protein expression in freshly isolated cells

**Figure 1:**
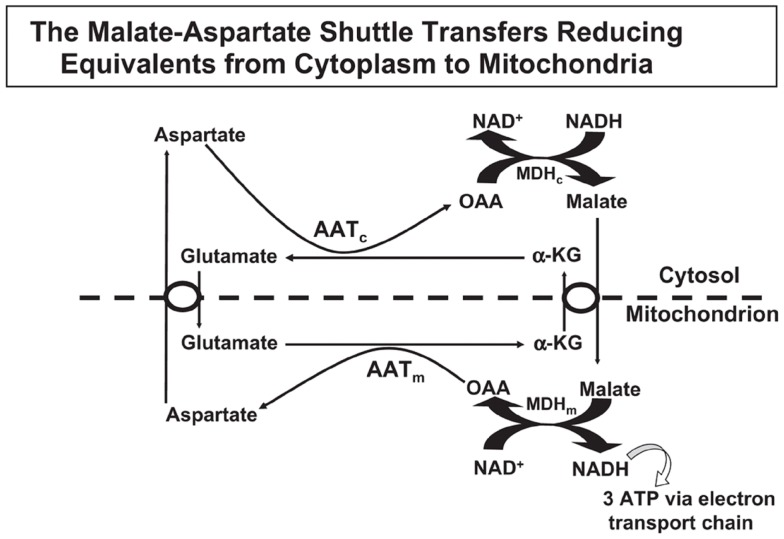
**In the malate–aspartate shuttle (MAS) cytosolic malate dehydrogenase (MDHc) oxidizes NADH and converts oxaloacetate (OAA) to malate (top right of figure), which enters the mitochondria in exchange with α-ketoglutarate (α-KG)**. The mitochondrial malate dehydrogenase (MDHm) re-oxidizes malate to OAA, which is transaminated to aspartate by the mitochondrial aspartate aminotransferase (AATm). Aspartate leaves the mitochondria in exchange with glutamate, requiring ACG (aralar or citrin). In the mitochondria glutamate conversion to α-KG is essential for AATm activity forming aspartate from OAA and delivering α-KG for mitochondrial export. The glutamate imported into the mitochondria had been formed by cytosolic aspartate aminotransferase (AATc) from α-KG after its entry into the cytosol. Without MAS activity NADH formed in the cytosol during glycolysis would have been unable to enter the mitochondria for oxidation. Reprinted from Hertz and Dienel (69), with permission.

Oxidative metabolism is needed by astrocytes for two major purposes, (i) to supply ATP for energy-consuming processes, and (ii) sto produce glutamate from glucose. This glutamate production is crucial for the production of transmitter glutamate and GABA, since glutamate does not easily enter the brain from the systemic circulation (55). The occurrence of one oxidative process during glycolysis, which generates NADH from NAD^+^ and the inability of NAD^+^ and NADH themselves to cross the mitochondrial membrane require that “reducing equivalents” are transferred across the mitochondrial membrane. Some shuttle mechanisms exist that are capable of doing this, but only the MAS is expressed in brain at a significant level as discussed by Dienel and Hertz (56). In the cytosol, reduction of oxaloacetate (OAA) to malate enables cytosolic NADH oxidation to NAD^+^, and malate can be transferred across the mitochondrial membrane for re-oxidation (Figure 1). In MAS it enters the mitochondria in exchange with α-ketoglutarate (α-KG), using the malate/α-ketoglutarate exchanger (OGC – *Slc25a11*). OGC expression is similar in synaptic and non-synaptic mitochondria (12), it is functional in astrocytes ((13) – see also Pardo et al. in this research Topic), and OGC is expressed in freshly isolated astrocytes (6). After its re-oxidation in the tricarboxylic acid (TCA) cycle to OAA, the latter becomes transaminated to the corresponding amino-acid, aspartate. Aspartate can exit across the mitochondrial membrane in exchange with glutamate, using the glutamate/aspartate exchanger (AGC). After its arrival in the cytosol glutamate is converted to OAA, closing the circle. Both mitochondrial and cytosolic aspartate aminotransferases (57–59) in brain are well established, and aspartate aminotransferase activity is high in cultured astrocytes (60, 61). Nevertheless, histochemistry failed to show expression of the enzyme by histochemistry in some studies (62, 63), although moderate astrocytic expression had previously been shown (64). This is peculiar in light of unhindered demonstration by the same authors of the expression of glutamate dehydrogenase (GDH), a mitochondrial enzyme which also metabolizes glutamate (63). Both the cytosolic (*Got1*) and the mitochondrial (*Got2*) aspartate aminotransferase gene have been demonstrated in freshly isolated astrocytes (4, 6).

**Figure 2:**
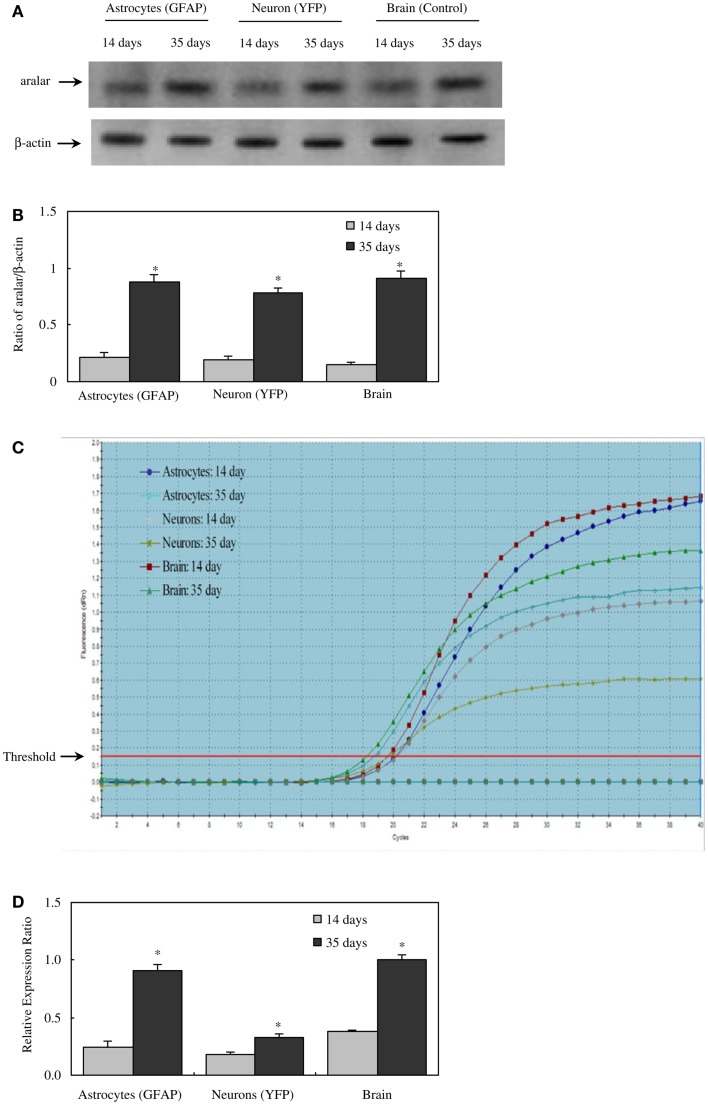
**Protein and mRNA expression of aralar in astrocytes and neurons isolated by FACS from cerebral hemispheres of 14- and 35-day-old astrocyte-labeled [F VB/NTg(GFAP-GFP)14Mes/J] or neuron-labeled [B6.Cg-Tg(Thy1-YFPH)2Jrs/J] mice and in intact brain of adult CD-1 mice**. **(A)** A representative immunoblot showing protein expression for aralar and β-actin, used as a house-keeping protein. The size of aralar is 70 kDa, and of β-actin 46 kDa. Similar results were obtained from three independent experiments. **(B)** Means ± SEM of scanned ratios between aralar and β-actin. *Statistically significant (*P* < 0.05) difference from the same preparation from 14-day-old animals. **(C)** A representative amplification plot of aralar mRNA expression, determined by real-time PCR. Similar results were obtained from three independent experiments. For analysis of graph, see Xu et al., under revision. **(D)** Means ± SEM (*n* = 3) of the relative expression ratio of aralar. *Statistically significant (*P* < 0.05) difference from the same preparation from 14-day-old animals. From (5).

There are two different AGC forms, in adult brain almost exclusively AG1 or aralar (*Slc25a12*), with only small clusters of citrin (*Slc25a13*) in a few neurons (65). MAS cycle activity is needed for formation of glutamate and GABA in brain, which is well known to occur readily and to depend upon pyruvate carboxylase-mediated (2, 66) glutamine formation in astrocytes. It therefore came as a big surprise when the operation of MAS in astrocytes *in situ* was questioned due to an observed absence of aralar (and citrin) in astrocytes *in situ* (11). This would make appropriate oxidative metabolism of glucose impossible. Ramos et al. reported that only little aralar expression is found in cultured astrocytes and even less in astrocytes of the adult brain. Berkich et al. (12) confirmed absence of astrocytic aralar using a different antibody. Finally, Pardo et al. (13), using an improved immunofluorescent assay with antigen retrieval and identifying astrocytes histologically, reported presence of aralar protein in astrocytes in the mouse brain, but only in relatively small amounts. However, in freshly isolated astrocytes and neurons obtained by fluorescence-activated cell separation (FACS) both microarray analysis (4) and RT-PCR (5) showed at least as much mRNA expression in adult astrocytes as in adult neurons, and the levels of expression were comparable to those in whole brain. Since expression of mRNA is not necessarily accompanied by protein expression, Li et al. (5) also determined protein expression of aralar, which was found to match its mRNA expression in cells from 35-day-old animals, whereas the expression was much lower in cells (both neurons and astrocytes) from 14-day-old animals (Figure 2). A similar slow development of aralar expression was shown in astrocyte cultures (Figure 2), and both the level of expression and the developmental course were similar in the freshly isolated cells and in the cultures used. Thus, there is no reason to doubt MAS function in astrocytes, although several immunohistochemical studies had indicated that this could not be the case.

The discrepancy between results obtained by IHC and FACS followed by RT-PCR and Western blot could be caused by: (i) use of GFAP to identify astrocytes in the studies by Ramos et al. (11) and Berkich et al. (12), since GFAP is absent from the fine processes that contain a large number of mitochondria (4); (ii) loss of antigenicity upon tissue fixation or tissue processing. That both points may be important is shown by the fact that Pardo et al. (13), the only authors who have demonstrated aralar immunohistochemically (i) relied on cell morphology, not GFAP presence to classify a cell as astrocytic, and (ii) also were the only ones to use antigen retrieval, a procedure that partly can overcome artifacts from fixation or tissue processing. Nevertheless, the consistency in the lack of ability to demonstrate aralar histochemically between several different groups underlines that this is not due to methodological errors in a single study, but to systematic, unexplained deficiencies within the methodologies. For some reason demonstration of the expression of many astrocytic genes is enigmatic.

#### Expression of genes of other enzymes involved in glucose metabolism

The discrepancy between results for aralar expression obtained by immunohistochemical analysis and by mRNA and protein determination raises the question whether a similar apparent failure of histochemistry to identify and accurately quantify expression of an important astrocytic gene also may apply to other genes. Lovatt et al. (4) carried out a microarray analysis of mRNA expression of a multitude of TCA cycle enzymes in FACS-isolated cells and found most of them to be expressed at higher levels in astrocytes than in neurons. In addition, these enzymes in freshly isolated astrocytes were functionally active, although to an undefined degree. However, Doyle et al. (10) reported much higher level of hexokinase gene expression in neurons than in astrocytes from bacterial artificial chromosome (BAC) transgenic mice, one of the newly established techniques (for more information see Fluorescence-Based Cell Sorting). This contrasts not only immunochemical studies (67), but also general concepts about energy metabolism in brain. Thus, Nehlig et al. (68) observed similar total glucose phosphorylation in astrocytes and neurons in intact brain tissue. Moreover, in the primary cultures used in our laboratory, the rate of glucose oxidative metabolism in astrocytes and cerebellar granule cells is quite similar, 1.2 and 1.0 nmol/mg protein per min [Table 6 in Ref. (69)], which is comparable with *in vivo* rates [reviewed by Ref. (70)]. One may therefore wonder if the animals used to study neuronal gene expression have shown more perfect labeling of their genome than those used for determination of astrocytically expressed genes. This should not conceal that many preparations of cultured astrocytes also show a very low rate of oxygen consumption (71), again emphasizing that not all astrocyte cultures are identical, and that many show characteristics making them unsuited as models for astrocytes *in vivo*. At least some enzymes involved in oxidative metabolism of glucose (and other substrates) have also been demonstrated immunohistochemically, since distinct astrocytic demonstration of cytochrome oxidase (COX) has been shown in sections from the monkey striate cortex (72). Astrocytes, but not neurons and probably also not oligodendrocytes show immunohistochemically determined expression of pyruvate carboxylase (2, 73), which is consistent with the operation of this enzyme in cultured astrocytes (66). The repeated demonstration of this enzyme in astrocytes is of crucial importance, since it is necessary for net synthesis of TCA cycle intermediates and thus for astrocytic production from α-KG of glutamate in astrocytes, needed for neuronal production of transmitter glutamate and GABA. Cultured astrocytes also express cytosolic, but not mitochondrial malic enzyme as shown both immunocytochemically (3) and by anion exchange chromatography to separate the cytosolic and mitochondrial isoforms of malic enzyme (74), whereas adult rat brain express the two isoforms about equally (suggesting neuronal localization of the mitochondrial form). There is consensus that cytosolic malic enzyme operates during complete oxidative metabolism of glutamate, converting it to pyruvate after its exit from the TCA cycle.

### Enzymes and transporters operating in glutamate and GABA turnover

Norenberg and Martinez-Hernandez (1) performed immunohistochemical analysis of GS in rat nervous system and showed that the enzyme was astrocyte-specific. The stain was confined to the cytoplasm and perivascular astrocytic processes. The intensity of staining varied between different brain locations, with highest level in hippocampus and cerebral cortex. GS expression has repeatedly been confirmed in astrocytic cultures [e.g., Ref. (75, 76)]. Presence of GS has also been claimed in oligodendrocytes in brain and spinal cord (77–79). However, oligodendrocytic manifestation was not described during the original demonstration of GS expression in astrocytes (1), and the absence of GS expression in oligodendrocytes has been confirmed by Derouiche (80) and again by Anlauf and Derouiche in the present Research Topic. The reader is referred to this paper for further discussion of this topic, which is important for understanding of both oligodendrocytic function and difficulties in correct demonstration of gene expression.

How glutamine transport is directed from astrocytes to neurons was long unknown, since no obvious differences were found between kinetics for glutamine uptake in astrocytes, cultured glutamatergic or GABAergic neurons, and neuronal perikarya prepared by gradient centrifugation (81). This problem has received its solution with the demonstration of different glutamine transporters in neurons and astrocytes. The bi-directional transporter SN1, also known as SNAT3 is abundantly expressed in astrocytic processes surrounding glutamatergic and GABAergic neurons and its expression is pronounced in the neocortex, cerebellum, olfactory bulb, and brain stem (82). The possibility that its absence from neuronal terminals could be due to insufficient antigen detection has been excluded by the demonstration that possible SN1/synaptophysin coexpression is rare (83). This system N transporter transfers glutamine in symport with Na^+^ and in antiport with H^+^, which is important for its role specifically in glutamine efflux from astrocytes (84). Its regulation by protein kinase C (PKC) is discussed in this Research Topic by Nissen-Meyer and Chaudhry. A potential role of its relative SN2 in some brain regions and its subcellular distribution have been discussed by Hamdani el et al. (85).

Released glutamate from glutamatergic neurons is mainly taken up by astrocytes ((86) – see also Zhou and Danbolt in this Research Topic), where it is either oxidative metabolized (∼20%) or converted to glutamine and re-transferred to neurons in the glutamine-glutamate cycle. Aspartate aminotransferase and/or GDH are involved in the conversion of glutamate to α-ketoglutarate. Aspartate aminotransferases were discussed above and astrocytic localization of GDH has been shown immunohistochemically by Aoki et al. (87) and Würdig and Kugler (63) and its gene expression has been demonstrated in FACS-sorted astrocytes by Lovatt et al. (4) and Cahoy et al. (6) using microarray analysis. It is reason for concern that astrocytic expression of this gene was not observed by Doyle et al. (10) in their study using BAC animals. In contrast, the genes shown to be expressed in Table 1 were as well recognized in the study by Doyle et al. (10) as in the two other microarray studies. Thus this methodology seems for unknown reasons to have difficulty demonstrating only the expression of certain astrocytic genes.

The astrocytically located cytosolic malic enzyme discussed above is responsible for α-ketoglutarate’s complete oxidation in the TCA cycle after conversion to malate and exit of malate to the cytosol. A larger fraction of released GABA than of released glutamate is re-accumulated in the neurons themselves but some is metabolized in the astrocytic TCA cycle after conversion to succinate via GABA transaminase (GABA-T) and succinic aldehyde dehydrogenase (SSADH) (see paper in this Research Topic by Schousboe et al.). GABA-T has been demonstrated histochemically in both neurons and astrocytes (88), consistent with GABA uptake in both cell types.

Among the five subtypes of glutamate transporters (excitatory amino-acid transporters; EAATs 1–5), astrocytes express l-glutamate/l-aspartate transporter (GLAST; EAATl) and GLT-l (EAAT2). Lehre et al. (15) and Chaudhry et al. (16) stained brain slices for GLAST protein and concluded it was expressed in astrocytes. Later, Schmitt et al. (14) compared GLAST mRNA and protein expression in the CNS of rat. They found that GLAST mRNA was located in the cytoplasm of astrocytes. In Bergmann cells, GLAST mRNA stain also appeared in proximal processes. Protein stain showed similar pattern, but fine processes that were not labeled with GFAP were also stained for GLAST. In the retina GLAST expression even in the finest Müller cell processes had previously been shown by Derouiche and Rauen (17). GLAST expression varies in astrocytes in different brain regions. In cerebellum, Bergmann cells showed strong reaction, but the granule cell layer showed only faint astrocytic labeling (14). GLT-1 is expressed in astrocytes of the mature brain and spinal cord (19, 20). Although GLAST expression is distributed within all cortical layers, and strongly expressed throughout the granule cell layer of the dentate gyrus of the hippocampus, experiments with a GLT-1-preferring inhibitor, WAY-855 showed that GLT-1 was responsible for 80% glutamate uptake in isolated hippocampal tissue (89). Selective inhibition of GLT-1 with WAY-855 does not completely prevent glutamate build-up, but NMDA receptor-mediated neurotoxic effects remain, suggesting additional roles of other glutamate transporters in extracellular glutamate maintenance. However, an authoritative review by Danbolt (86) has also concluded that GLT-1 is the dominant glutamate transporter in cortical astrocytes and that GLT-1 and GLAST together account for the predominant astrocytic uptake of glutamate in the brain *in vivo*.

Kinetics for glutamate uptake has been determined by different authors in primary cultures of astrocytes, where the uptake can be extremely intense (90). Swanson et al. (91) and Schlag et al. (22) found that primary cultures of rat astrocytes express GLAST, but little or no GLT-1, and that treatment with dBcAMP, known to increase intracellular cAMP, enhanced the expression of both GLAST and GLT-l. This observation was made by both ICC and mRNA and protein determination. Nevertheless, GLT-1 expression was only a fraction of that observed in brain tissue (22). Co-culturing with neurons had a similar effect as the cAMP analog. dBcAMP treatment also increased *V*_max_ for glutamate uptake (22), although it only reached about one half of its value in mouse cultures found by Hertz et al. (90). GLT-1 expression in cultured astrocytes can also be enhanced by activation of additional signaling pathways (23), and it would be extremely useful to establish an astrocyte culture with as high GLT-1 expression as in the brain *in vivo*.

Some released transmitter GABA is taken up by astrocytes, although the uptake rate in cultured astrocytes is much slower than that of glutamate (92) and also slower than in cultured neurons (93). GABA uptake is mediated by one of three high-affinity subtypes of GABA transporters (GATs), GATl, GAT2, and GAT3 or in some cases by the one low-affinity transporter BGTl. Astrocytes can express all four subtypes [for review, see Ref. (25)]. GAT1 is present in astrocytes in all brain regions, where IHC demonstrated punctate structures that were shown by electron microscopy to be located exclusively in the small cell processes (26). In cerebellum, GAT1 mRNA was detected in Bergmann cells [for review, see Ref. (25)]. In rat thalamus GAT1 and GAT3 proteins are mainly expressed in astrocytes and the stain of GAT3 is more intense than that of GAT1 (28). In the parabrachial and Kölliker-Fuse nuclei GAT3 was detected, whereas GAT1 was absent (29). Cerebellar mRNA and protein staining of GAT2 and GAT3 was also primarily glial, with GAT2 stain in the granule layer and GAT3 stain in the deep nuclei (24, 27). Nevertheless, FACS isolation followed by microarray analysis showed the GAT2 gene as not expressed in brain (6, 9) a similar result was reached in cerebellum using BAC transgenic mice (10). With respect to most other glutamate and GABA transporters there is virtual consensus by all methods that they are expressed in astrocytes. An exception is BGT-1 mRNA, which is observable in cerebellum of BAC transgenic mice (Table 1), although not with sufficiently high fold change for unequivocal demonstration (10). It has also has been reported in cultured astrocytes (30), although up-regulation of this betaine-GABA transporter did not affect GABA uptake (31).

### Nucleoside transporters and adenosine kinase

There are two types of nucleoside transporters, the concentrative CNTs l–3, which are able to transport nucleosides against an intracellular/extracellular gradient of the nucleoside itself, and the equilibrative ENTs l–4. CNTs are necessary for termination of adenosine and guanosine transmitter effects by cellular uptake, regardless of cellular requirements. ENTs, like other equilibrative transporters, carry out transport only until equilibrium has been established between intra- and extracellular concentrations (94). Most nucleoside transporters are membrane-bound, but ENT3 is mainly intracellular (33), and may distribute adenosine into intracellular organelles (34).

**Figure 3:**
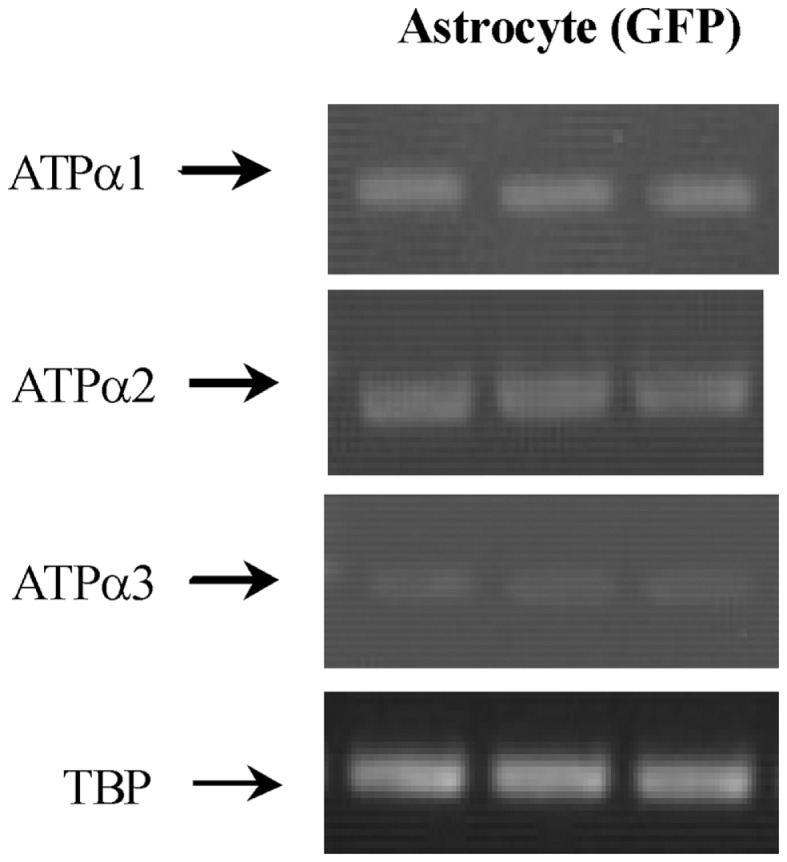
**mRNA expression measured by RT-PCR of α1, α2, and α3 isoform of Na,K-ATPase in astrocytes isolated by FACS from cerebral hemispheres *in vivo* from adult mice [FVB/NTg(GFAP-GFP)14Mes/J]**. A representative experiment showing mRNAs for α1, α2, and α3 isoform and for TBP, as a house-keeping gene. The sizes of the PCR products of α1 is 920 bp, α2 350 bp, α3 329 bp, and TBP 236 bp, *n* = 3. From (43).

mRNA expression of ENT1 and ENT2 mRNA, determined by ISH, is widespread in the brain (32), and the presence of both of these transporters has been reported in both astrocytes and neurons in a review by Parkinson et al. (33). The same review claimed that CNT2 and CNT3 were absent in astrocytes, and no information was provided about ENTs 3 and 4. Moreover, the impression was given that astrocyte expression of the remaining transporters was low. However, this information seems to be misleading, since mRNA for all nucleoside transporters except CNT1 are expressed to at least a similar degree (in relation to applied amount of RNA and to a house-keeping gene) in astrocytes as in neurons in freshly isolated astrocytes (34) (Table 1). Analysis using either FACS-sorted cells or BAC transgenic animals also demonstrates most of them although with some differences between different investigators and especially good recognition by Doyle et al. (10). The astrocytic enrichment in ENT3 was dramatic (Figure 3), opening the possibility that gliotransmitter ATP may be synthesized in intracellular organelles, which has been confirmed by downregulation of ENT3 by siRNA (Xu et al., under revision). It is consistent with intense astrocytic but not neuronal formation of AMP, ADP, and ATP in cultured cells (95) that adenosine kinase, the enzyme converting adenosine to AMP in the adult brain, has been found by IHC to be selectively expressed in astrocytes (96). However, Parkinson et al. (97) reported very little nucleotide formation in astrocyte cultures, perhaps reflecting immaturity of the cells used, and again emphasizing that different astrocyte cultures may behave differently. As illustrated for ENT3 in Figure 3, the expression of ENTs and CNTs shown in freshly isolated cells has been replicated in our cultured cells (35, 43) with the exception of CNT3, which is sparsely expressed in brain.

### Expression of genes of transporters involved in K^+^ clearance from the extracellular space

Two astrocytic membrane proteins mediate uptake of extracellular K^+^ ([K^+^]_e_), Na,K-ATPase and NKCCl, which is an inwardly directed Na^+^, K^+^, 2Cl^−^ cotransporter expressed both in cultured astrocytes (98–100) and mature astrocytes *in vivo* (101, 102). The Na,K-ATPase operates alone below a total [K^+^]_e_ of ∼10 mM. At higher [K^+^]_e_, NKCC1 plays a dominant role, as shown by inhibition with bumetanide or furosemide, inhibitors of the cotransporter (103–105).

Na, K-ATPase contain α and β subunits. Three isoforms of its α subunits (α1–3) and 2 of its β subunits β1 and β2) are expressed in brain (38, 39). Immunofluorescent histochemistry showed that ATPase α1 protein is expressed both in neurons and glia, and its glial expression is obvious in co-cultures (41). Only Cahoy et al. (6) indicate expression of α1 mRNA after microarray analysis in spite of its demonstration by histochemistry, ISH (in cultures) and also after FACS separation followed by RT-PCR (43). The failure of both Lovatt et al. (4) and Doyle et al. (10) to show astrocytic expression of this gene in, respectively FACS-isolated astrocytes and astrocytes obtained from BAC mice treated with the astrocytic marker Aldh1L1 is unfortunate.

Na, K-ATPase α2 expression is primarily in glia, and Na,K-ATPase α3 is only expressed in neurons (40, 44). Expression of α2 in astrocytes was first suggested by Watts et al. (42), who demonstrated its mRNA expression with ISH. Its labeling pattern was diffuse in hippocampus, neocortex, and brain stem. Later, Sweadner’s group performed IHC on cerebellar brain slices and ICC on co-cultures of cerebellar granule cells and astrocytes (41) and consolidated their findings by ISH. Staining for α2 in the granular layer was observed in diffuse processes around granule cells (41) in a similar staining pattern as that found by McGrail et al. (40). In the co-cultures α2 staining was extensive in GFAP-positive astrocytes and it uniformly labeled the surface of the cells (41). Recent experiments using cells obtained by FACS have confirmed α2 expression in astrocytes (Figure 3) (43) in agreement with the previous observation by IHC, ICC, ISH, and cell culturing. Microassay analysis (Table 1) has also consistently shown expression of the gene of the α2 isoform of the Na,K-ATPase in astrocytes (4, 6, 10).

*In situ* hybridization showed NKCC1 mRNA located in granule layer and white matter tract of cerebellum (45). Attempts to demonstrate NKCC1 immunohistochemically in astrocytes has, however, also often provided negative results (M. Nedergaard, personal communication). Nevertheless functional responses to high extracellular K^+^ concentrations (swelling or enhanced K^+^ uptake and their inhibition by a NKCC1 inhibitor) have repeatedly identified the astrocytic location of this transporter both in the superfused monkey brain (106) and cultured astrocytes (104). Gene expression and phosphorylation of NKCC1 have been studied in cultured astrocytes under control condition and after trauma by increased barometric pressure by Jayakumar et al. (107). Silencing of NKCCl with siRNA led to a reduction in trauma-induced NKCC1 activity as well as in cell swelling. Microarray results by both Cahoy et al. (6) and Doyle et al. (10), but not those by Lovatt et al. (4), also indicate its expression in astrocytes (Table 1).

### Comparison of results obtained by different methodologies

Expression of some, but far from all genes in astrocytes seem to be impossible to demonstrate or quantitate with IHC and ISH (Table 1), even using optimized techniques, whereas their expression is clearly shown in intact cells freshly obtained from the brain. Biochemical analysis of these cells (determination of mRNA or protein) gives somewhat more consistent results than microarray analysis. There is no systematic explanation for the failure of IHC and ISH to show expression of certain genes, and the genes in question can be located either on the cell membrane (most of the nucleoside transporters) or intracellularly (aralar and ENT3). Results obtained by biochemical analysis in cultured astrocytes (see below) can be very similar to those obtained *in vivo*, but unfortunately great differences are found between results obtained in different cultures. The recently introduced BAC transgenic mice selecting genes co-expressed with the astrocyte-specific *Aldh1L1* gene seem also to have severe difficulties in recognizing several astrocytically expressed genes. There are no overlapping methodological mechanisms between this technique and IHC, but there might some with ISH, perhaps helping to answer the question why demonstration of specifically astrocytically expressed genes is so enigmatic. It would also be interesting to compare astrocytic gene expression determined in BAC mice selected with a different astrocyte-specific gene.

## Description of Methodologies Used for Determination of Gene Expression

### Immunohistochemistry and immunocytochemistry

By determining the expression of protein, IHC and ICC should be the ideal tools for demonstrating gene expression in different cell types. IHC is a laboratory technique that uses specific antibodies to detect antigens in cells of a tissue section. ICC uses samples of intact cells without their surrounding extracellular matrix and contacts to other cells of different types, and is used for many types of cultured cells. The immunohistochemical study that did demonstrate aralar (13) used a polyclonal antibody, Ramos et al. (11) a previously prepared antibody by the same group, and Berkich et al. (12) a donated antibody, recognizing both citrin and aralar. In general, the binding site of the antibody is unknown except when a synthesized peptide is used as the antigen. Fixation is necessary and also difficult. Formaldehyde is the gold standard for fixation. Its basic mechanism is to form a product between the formalin and uncharged reactive amino groups (-NH or -NH2) by aid of cross-links and eventually change the three-dimensional structure of the proteins [for review, see Ref. (108)]. Overfixation produces false negative results. Antigen retrieval is usually used to reverse this conformation change, and among the papers studying aralar expression immunohistochemically it was used by Pardo et al. (13), but not in the other publications. In brain slices, GFAP is generally used as astrocytic marker, for example by Berkich et al. (12), but not by Pardo et al. (13), who relied on morphological characteristics. One problem is that GFAP is a cytoskeleton protein that is rarely distinguishable in all astrocytes (generally more after cell damage) and that its staining may not be representative of cytosolic and membrane proteins. Even when expressed in an astrocyte, GFAP is not present in the peripheral processes (4, 109) but only expressed in the main stem branching branches, and often even absent in the cell body. Using GFAP as a marker in IHC one may therefore significantly underestimate the abundance of genes of interest expressed in astrocytes (109). Many other genes and the proteins they code for are astrocyte-specific in adult animals, including GS, excitatory amino-acid transporter 2 (EAAT 2), and its rodent analog GLT-1, aquaporin 4, connexin 43 (ctx43), ALDH1L1 (6, 110) as well as the epithelial Na^+^/H^+^ exchanger regulatory factors NHERF1, and GLAST (EAAT-1) (111), with the latter showing pronounced species variability in the expression of its splice variants (112). An excellent marker for astrocytes seems to be EGFP introduced into transgenic mice under the control of the human GFAP promoter (7). This reporter molecule is a much better astrocytic marker than GFAP, since GFP is expressed even in the fine processes. GS is another astrocyte marker which is much better than GFAP (Anlauf and Derouiche, this Research Project). Thus, it may be possible to improve immunohistochemical demonstration of astrocytically expressed genes by discontinuing the use of GFAP as an astrocytic marker and rely more on other markers and/or morphology. The presence of antigen labeling present predominantly in the fine peripheral astrocyte processes, e.g., ezrin (113) and reelin may appear as a hazy “background.” This can easily be overlooked, regardless which astrocyte-specific gene is used for cell identification and should be carefully checked for, although this can be difficult in intact brain.

### *In situ* hybridization

The ISH technique is performed in fixed tissue with all cellular relationships remaining intact. It determines anatomic localization of labeled or non-labeled RNA or DNA probes that hybridize to target complementary RNA or DNA sequences in the cell (114). These hybrids can be detected using either an isotopic probe and emulsion autoradiography or non-isotopic methods using specific antibodies to detect a hapten incorporated into the probe. Radioisotopic ISH is perceived as providing high sensitivity, quantitative labeling, and relatively unambiguous discrimination of signal versus background, whereas non-radioactive colorimetric ISH often provides better anatomic localization and discrimination between cells and is well suited to produce large amounts of data (114). ISH is limited by providing no information about translation and posttranslational processes on its own. This obviously also applies for expression of mRNA determined by different methodologies, for example FACS and BAC analysis. Taking advantage of automated high-throughput procedures and data acquisition ISH has been used to generate a digital atlas (the freely available Allen Brain Atlas) showing the expression patterns of approximately 20,000 genes in the adult mouse (115). Potential failure to identify expression of some astrocytic genes in an unpredictable manner in this excellent standard reference work would be most regrettable. Information in general about correlation between protein and mRNA expression would be valuable and an overall estimate of the frequency of differences would therefore be very valuable.

Several groups have conducted mRNA/protein correlation analyses, but the results are controversial. Guo et al. (116) carried out a correlation study for 71 genes using human circulating monocytes and showed an overall positive correlation. Gry et al. (117) investigated 23 human cell lines and 1066 genes and found that only one third of the genes showed significant correlation between mRNA and protein expression. Schwanhäusser et al. (118) found better correlation between mRNA and protein levels than previously thought.

## Cell Separation Techniques, BAC Transgenic Mice, and Methodology for mRNA and Protein Determination

### Microdissection and gradient centrifugation

Using the large Deiters’ cell neurons and surrounding neuropil as well as microanalytic techniques Holger Hydén demonstrated more than 50 years ago that learning changes the base composition of nuclear RNA, in both neurons and glia. He also established that glial cells show more marked and earlier changes in RNA composition in Parkinson’s disease than neurons. He had the vision and courage to suggest that “mental diseases could as well be thought to depend upon a disturbance of processes in glia cells as in the nerve cells,” and showed that antidepressant drugs cause profound changes in glial RNA [for review, see Ref. (119, 120)].

There is no doubt that the neuropil samples constituted less pure glial samples than present-day astrocytic preparations, but nevertheless many of Hydén’s observations are presently being confirmed. A different technique to separate neurons and glia, gradient centrifugation (121) was used in his laboratory by Hamberger and coworkers to show glial uptake of glutamate and GABA for the first time and many features of their subsequent metabolic fate (122, 123). Such studies are suitable for determination of *K*_m_ values, but the cells are too damaged to evaluate *V*_max_.

### RT-PCR following electrophysiological selection of astrocytes with specific receptor expression

The Steinhäuser group has carried out a series of studies combining patch clamp analysis with RT-PCR in studies of AMPA receptor subunit expression in astrocytes obtained from hippocampal slices (124–126). Facilitated by previous treatment with enzymes, glial cells expressing AMPA receptors were extracted after identification based on electrophysiological and immunocytochemical properties, including absence of action potentials. At the end of the recording, a negative pressure was applied to the pipette sucking in the cell, the tip of the pipette was broken off and the cell’s contents were harvested under a microscope as originally described for Purkinje cells by Lambolez et al. (127). mRNA expression in the collected cells was analyzed with RT-PCR using repeated amplification and with emphasis on determination of AMPA receptor subtypes and astrocyte-specific compounds. The observed changes in splice variant expression and subunit assembly of AMPA receptors during cell maturation (125) is to be expected due to late generation of astrocytes (128) and profound alterations in gene expression and function in the mouse/rat brain during the first three postnatal weeks (129, 130).

### Cell sorting based recognition of cell-specific proteins

#### Fluorescence-based cell sorting

The possibility to associate a specifically fluorescent drug to either neurons or astrocytes via their genetic promoters (7) and subsequently sort cells freshly dissociated from the brain according to their fluorescent characteristics can greatly improve the purity of the obtained cell fractions. While Fluorescence-activated cell sorting (FACS) has long been used in the immunology and cancer fields, its use in neuroscience was until recently limited to embryonic brain tissue, cultured cells, stem cells, or synaptosomes, because these cells or organelles lack or have fewer processes than adult neurons and astrocytes (131). Pioneering studies in Maiken Nedergaard’s and Ben Barres’s laboratories established this technique for use in adult (4) and adolescent (6) brain.

A genetically transformed (GFAP-S65T-GFP) mouse was originally generated by Zhuo et al. (132) from the Messsing group by inserting a 2.1 kb DNA fragment of the human GFAP promoter randomly into the mouse genome during oocyte injection of linearized transgene DNA for transgene generation. In many current references this mouse is called a GFAP-GFP or even GFAP-EGFP mouse, but it is a GFAP-S65T-GFP mouse. S65T-GFP is a modified and brighter fluorescent protein than the wild type GFP, but it is not as bright as EGFP (enhanced GFP) (7). The Nedergaard laboratory (4) used this mouse in combination with immunohistochemical labeling with anti GLT-1 antibodies to label astrocytes in adult mice for sorting by FACS. The combination of the two labels increases the purity of the isolated astrocytes.

Dissociated cells from the cerebral hemispheres of such mice were sorted and collected by a cell sorting system according to the wavelength of their fluorescent signal. Cell purity has been determined by mRNA expression of cell markers of astrocytes (*Gjb6, Gfap, Slc1a2, and Fgfr3*), and lack of mRNA from markers of neurons (*Gabra-l, Slc12a5, Snap25*, and *Syt1*), and oligodendrocytes (*Gjc2, Mag, Mog, and Mbp*). A relatively small number of cells (∼8%) die (become PI^+^-positive) as a result of the procedure. Metabolic activity has been demonstrated in similar cells (4), but not quantitated. The astrocyte sample yield from FACS is about 1–2 μg RNA or 20 μg protein per brain which is sufficient for a multitude of microarray assays (4, 6) or for ∼10 RT-PCR determinations (5, 34, 43, 47, 133). Our aralar study (5) and further FACS studies were carried out using this technique, although without the GLT-1 antibodies, again with no contamination of the astrocyte samples with either neurons or oligodendrocytes (133). Our studies showed that determination of the expression of a moderate amount of genes (34, 43, 47) or of a single gene (5) by RT-PCR yields highly reproducible and comparable results, regardless whether “classical” RT-PCR or qRT-PCR (real-time RT-PCR) was used, as can be seen by comparison between Figures 2C and 2D. The Barres laboratory (6) used FACS combined with immunopanning to isolate astrocytes from transgenic mice that express EGFP under the control of an S100β promoter.

#### Cell sorting based on naturally expressed cell-specific genes

A FACS-like method that does not depend on the use of transgenic animals has been developed for isolation of neurons and endothelial cells by Guez-Barber et al. (131). Specific fluorescent labeling of neuronal cells was obtained by treating the cells obtained after brain dissociation with a biotinylated NeuN antibody and subsequently with phycoerythrin-labeled streptavidin. Streptavidin has an extremely high affinity for biotin and phycoerythrin is a protein that produces a bright red-orange fluorescence, allowing conventional FACS methodology to be used for these cells, selected by Neu1 expression, and made fluorescent by the subsequent binding of phycoerythrin-labeled streptavidin. Jungblut et al. (134) used a related approach to obtain astrocytes from young postnatal brain. Their cell suspension was first labeled with the anti-GLAST antibody ACSA-1, conjugated to biotin, whereupon superparamagnetic MicroBeads coupled to an anti-biotin antibody were applied. The cells were resuspended in PBS with 0.5% BSA and the cell suspension was loaded onto an MS column (Miltenyi Biotec), which was placed in the magnetic field of a MiniMACSTM Separator from the same company. The magnetically labeled GLAST^+^ cells were retained within the column and eluted after removing the column from the magnet. Viability of the cells was demonstrated by subsequent culturing, showing proliferation and the formation of a dense layer of GLAST/GFAP double-positive cells. However, the usefulness of the present modification is limited by the fact that astrocytes could only be successfully isolated from P1–P10 mice. When older animals were used, the presence of cell debris after tissue dissociation interfered with the separation performance, lowered the purity of the isolated cells, and diminished their viability by at least 20%. Use of cells from very young animals can be gravely misleading because of important postnatal changes (129, 130).

#### Cell sorting using bacterial artificial chromosome transgenic mice

Heiman et al. (135) from the Heintz laboratory generated transgenic mice that expressed the ribosomal protein L10a tagged with EGFP. Using these mice one can achieve proper cell-specific labeling by green fluorescence and simultaneously use anti-GFP antibodies for immunopurification of ribosomes. Since the ribosomes carry mRNA for translation, mRNAs that are currently translated are co-purified. Accordingly, brains from mice of any age can be obtained and homogenized, and passage of the homogenate through a column with anti-GFP antibodies or immunoprecipitation will provide the currently translated mRNA in the cell type studied. In contrast to the FACS/immunopanning approaches cell viability is of no concern.

For their study of astrocytic gene expression Doyle et al. (10), also from the Heintz group, did not use the human GFAP promoter (as had been done in the Messing laboratory) but instead used a different astrocyte-specific (6, 110) marker, *Aldh1L1*. For proper transgenic labeling they used long stretches of genomic DNA (up to 100 MB in size), which can only be manipulated and amplified as BACs. In these DNA fragments the original gene is present, with probably all elements required for proper gene expression. When the open reading frame is replaced by EGFP, fluorescent cells are obtained [as in the study by Lovatt et al. (4)], and when it is replaced with L10a-EGFP ribosomes with adhering mRNA are obtained. Like the much shorter GFAP-EGFP construct the long BAC construct is injected into mouse oocyte for transgene generation. BAC transgenic mice have become popular, since there is a huge repository with BACs for almost all genes. In addition, there is a huge collection of transgenic mice generated by this approach (see www.gensat.org). The general assumption in the field is that BAC transgenes are more specifically expressed than the shorter promoter-using transgene constructs. For many cases, this is true, but there are also exceptions. *Aldh1L1* is a rather universal astrocytic marker, and it is almost unthinkable that *Aldh1L1-*expressing cells should not express the α1 unit of the Na,K-ATPase or GDH. It is therefore surprising (and disappointing) that genes for these proteins were not recognized in the study by Doyle et al. (10). Future studies of astrocytic gene expression using BAC mice will determine the general validity of this method for determination of astrocytic gene expression.

#### Direct mRNA sequencing

A newly developed technique, direct mRNA sequencing (DMS) (136), allows use of much smaller amounts and is well suited for determination of gene expression in astrocytic subcompartments (137). Like the methodologies used by Lovatt et al. (4) and Cahoy et al. (6) it requires initial isolation of the cells and the subcompartments to be investigated. It has generally been used together with microarray analysis but could also be used for RT-PCR (137) and thus enable accurate mRNA determination using smaller amounts of cells.

#### Evaluation of the microarray data

The studies by Lovatt et al. (4), Cahoy et al. (6), and Doyle et al. (10) all investigated astrocytic gene expression, although based on different astrocyte-specific genes. All used similar microarray procedures, but different sensitivities and purities are intrinsic to all of these methods. Most of the results probably overlap for up to 80% (see also Table 1), but particularly mRNAs with low abundance might be not detected in one or the other technique. However, the evasive nature of determination of astrocytic gene expression seems also occasionally apply to these newer methods. Among the relatively few genes shown in Table 1, expression of the aralar gene (*Slc25a12*) was not recognized in the study by Doyle et al. (10), which is worrisome. The lack of demonstration of expression of genes for ENT2 in the Doyle study and for ENT3 in the Lovatt study (in spite of its strong expression, determined by RT-PCR in both FACS-isolated cells and cultured cells shown in the present communication) is also reason for concern. Part of the problem might be the innate uncertainty of the microarray analysis, a problem that is obviously not solved by enrollment of additional gene identification methods. This concept is supported by results from Table 2, showing identical gene expression and editing determined in FACS-isolated and cultured astrocytes. However, expression of many of the same genes were not recognized by Lovatt et al. (4), using similar isolation procedure but determination of gene expression by microarray analysis. This applied also to the related studies by Cahoy et al. (6) and Doyle et al. (10), which also used microarray analysis. The safest procedure seems accordingly to be study of the expression of only one or a few genes of immediate interest and use of enough material (if needed, several animals) for an analysis by RT-PCR, repeated microarray analysis, or even better determination of protein by reaction with antibodies. Generally FACS-separated cells are not used for protein determination by Western blot and subsequent reaction with a specific antibody [only exception: aralar determined by (5)], since determination of multiple antigens (gene under study and house-keeping gene) by Western blot requires sample sizes between 25 and 75 μg protein (with some variability between the antigens of interest) for each determination. A single protein determination may therefore require brains from more than one animal.

### Primary cultures of astrocytes

The pioneering technique of Booher and Sensenbrenner (138), allowing easy preparation of cerebral astrocytes, led to experiments providing many of the first hints of astrocytic characteristics and gene expression. A wealth of information was obtained, some of which stands, whereas others were shown to be incorrect, at least partly dependent upon the culturing method used. The value of cell culture studies in many different areas of astrocytic biology and pathology has been authoritatively reviewed by Lange et al. (139). The final version of our own cell cultures dates from 1978 (90). These cells are prepared from newborn male or female mice, and the methodology and functional characteristics have been described in some detail by Juurlink and Hertz (53), Hertz et al. (140), and Hertz (141).

The cultures can also be prepared from rats, but the rat astrocyte cultures are more contaminated with other cell types (41), and other differences, including much lower unidirectional K^+^ influx (reduced membrane conductance) have also been reported (41, 54) and remain unexplained. From the age of 14 days, 0.25 mM dBcAMP is included in the medium. This compound increases intracellular cyclic AMP and promotes differentiation in astrocyte cultures derived from newborn brain (142–144). The age of 2 weeks for its addition has been determined experimentally. This is consistent with the finding by Moonen and Sensenbrenner (145) that astrocytes need a certain stage of development in order to respond to dBcAMP, and that by Lodin et al. (146) that astrocytes de-differentiate *in vitro*, unless treated with this compound. Close similarities between the presently used cultures and freshly isolated astrocytes in not only levels of aralar protein and mRNA expression (5) but also rates of developmental changes support their validity as models of their *in vivo* counterparts. The same applies to gene induction following treatment with antidepressant or antibipolar disorder and their effects on not only gene up-regulation but also gene editing (47, 48), shown in Table 2. Neuronal cells are absent in these cultures. They contain <1% of non-parenchymal brain cells (e.g., meningeal or endothelial cells), a very small number of microglia (3%) and 95% of the cells are positive for GFAP and for GS (147). In contrast to the cells cultured by Foo et al. (50) from the astrocytic cell fraction obtained by their FACS technique from 1-week-old animals, our cultures survive well without growth factor addition to the media, but serum is routinely present, although at reduced amounts during later culturing stages. Foo et al. (50) successfully cultured the cells they obtained and showed that gene expression in the cultured cells mimicked that in FACS-isolated cells to a greater extent than the cultured cells (quite different from our cultured cells) they had previously studied (6).

There are many reasons that all cultured astrocytes are not identical. Differences in procedures used for preparation of astrocyte cultures by different authors include species (rats or mice), dissociation methods, amount of cells seeded, and medium used, but most procedures use new-borne mice or rats. That our cultures are so highly enriched in astrocytes that they require no subsequent cell separation, may be related to the small number of cells seeded and the small pore size used for filtering the cells. However, the most important difference between our astrocyte cultures and those used by other investigators is probably the addition of dBcAMP from the age of 2 weeks. One reason that we have chosen to supplement the culturing medium with dBcAMP is that most noradrenergic innervation from locus coeruleus (to a large extent contacting astrocytes) is reaching the forebrain at the time of birth (148). Since this is at the time the cells are harvested for culturing, they have not received the noradrenergic input which is important for brain development *in vivo* [e.g., Ref. (149)]. One may wonder if one of the reasons the astrocyte cultures established by Foo et al. (50) from 1-week-old astrocytes mimic astrocytes *in vivo* better than the cultured cells with which they were prepared is that they had received noradrenergic signaling at the onset of culturing. dBcAMP greatly enhances many characteristics, including gene expression of aralar (Figure 2). Expression of several proteins are altered (142, 143). In many cases it is obviously difficult to show that these changes make the cells more astrocytic, and many astrocytic features develop also without dBcAMP treatment. However, one important characteristic is that in the absence of dBcAMP treatment elevated concentrations of K^+^ fail to stimulate glycogenolysis, a trait typical for astrocytes in normal brain tissue (52), whereas they do so after treatment with dBcAMP (51). dBcAMP was also found to increase the expression of GLT-1, but not to its level in brain. A goal of future research might be to establish similar GLT-1 gene expression in the cultures as in astrocytes in the brain *in vivo*. Increased use can be encouraged of good cultured cells, which by comparison with appropriately analyzed cell fractions have repeatedly shown to provide reliable results.

### Summary

The expression of some astrocytic genes both in freshly isolated cells and in cultured astrocytes is undoubtedly real as shown by its repeated demonstration in different preparations using different techniques. Isolated preparations of astrocytes provide very useful information, but the intricate anatomic connections in adult brain represent a formidable barrier for preparation of freshly dissociated astrocytes from the adult brain in sufficient amounts. In spite of continuous progress in analytical methods, which may also decrease the amount of cellular material needed, the “bottleneck” for use of freshly isolated cells from adult animals by two different methods (4, 6) remains the lack of inexpensive and very gentle dissociation methods, preferably yielding large amounts of cells. Some, but not all, preparations of cultured astrocytes have shown themselves to closely resemble freshly isolated astrocytes in the expression of a multitude of genes. They have provided crucial information and appear also to be usable for further studies. The developmental signals to the cultures delivered by noradrenergic stimulation may be important determinants of their “astrocyticity.” Nevertheless, they also have room for further improvement.

## Conclusion

Expression of many, but not all astrocytic genes can be appropriately recognized, at least qualitatively, by IHC and ISH. However, there are very important exceptions. The deficient aralar expression is probably no longer of major concern, since it has been contradicted by studies in both freshly dissociated astrocytes and in cultured cells. Moreover, lack of aralar expression is inconsistent with known astrocytic functions. The deficient qualitative and in some case quantitative expression of nucleoside transporters presently seems to represent a much bigger problem, requiring additional studies and changes of current concepts. Alternative methods may also fail. It is thus unfortunate that cell separation followed by microarray analysis in several cases provide different results in different studies and show negative results even when FACS sorting followed by determination of gene expression by RT-PCR and even IHC or ISH have indicated expression of a specific gene. Development of cheaper and gentler cell separation techniques is also urgently needed, but is bypassed when the method by Doyle et al. (10) is used. Gene expression determined in cultured cells can be reliable, but the cells used should have proven themselves capable of showing characteristics consistent with those of astrocytes obtained by other methodologies [Table 2; (50)]. The fact that several of the astrocytic genes expressed both in freshly isolated and cultured cells are neither recognized by IHC or ISH, nor by the elegant newer cell-labeling techniques even in the hands of careful investigators is disturbing. It may be even worse that this seems to happen in a completely unpredictable manner. We have reviewed some of these genes but are in no doubt that expression of additional astrocytic genes may remain unrecognized.

## Conflict of Interest Statement

The authors declare that the research was conducted in the absence of any commercial or financial relationships that could be construed as a potential conflict of interest.

## Abbreviations

AGC, glutamate/aspartate exchanger; BAC, bacterial artificial chromosome; BGT, low-affinity GABA transporter; CNT, Concentrative nucleoside transporters; COX, cytochrome oxidase; DMS, direct mRNA sequencing; dBcAMP, dibutyryl cyclic AMP; EAAT, glutamate transporters; ENT, equilibrative nucleoside transporters; FACS, fluorescence-assisted cell sorting; Fgfr, fibroblast growth factor receptor; GATs, high-affinity subtypes GABA transporters; GFAP, glial fibrillary acidic protein; GS, glutamine synthetase; α-KG, α-ketoglutarate; MAS, malate-aspartate-shuttle; OAA, oxaloacetate; OGC, malate/α-ketoglutarate exchanger; PI, propidium iodide; RT-PCR, reverse transcription-polymerase chain reaction; TCA, tricarboxylic acid.
